# Evaluation of molecular model-based discovery of ecto-5’-nucleotidase inhibitors on the basis of X-ray structures

**DOI:** 10.1186/1758-2946-6-S1-P13

**Published:** 2014-03-11

**Authors:** Norbert Furtmann, Jürgen Bajorath

**Affiliations:** 1 Department of Life Science Informatics, B-IT, LIMES Program Unit Chemical Biology and Medicinal Chemistry, Rheinische Friedrich-Wilhelms-Universität, Dahlmannstrasse 2, D-53113 Bonn, Germany

## 

*Ecto*-5’-nucleotidase (e5NT) belongs to the family of metallophosphoesterases, hydrolyses AMP to adenosine, and is a regulator of the adenosine signaling pathway [1]. It has been shown, that free adenosine is involved in various diseases and cancer progression [2,3]. In a previous study, a molecular model of e5NT has been created and used for the identification of new sulfonamide inhibitors [4]. Recently, X-ray structures of human e5NT in complex with different inhibitors were published [5]. This made it possible to reevaluate the model building and virtual screening efforts in detail. An extensive analysis of the comparative e5NT model, built using a bacterial enzyme in the presence of 35% sequence identity as a template, showed that the model was topologically correct and had high accuracy within the active site region. Comparative docking studies were carried out to explore inhibitor binding characteristics within the X-ray structure and the model. The results provided plausible explanations for the successful identification of new e5NT inhibitors by model-based virtual screening and highlighted important parameters [6].
